# Collagen Metabolism of Human Osteoarthritic Articular Cartilage as Modulated by Bovine Collagen Hydrolysates

**DOI:** 10.1371/journal.pone.0053955

**Published:** 2013-01-16

**Authors:** Saskia Schadow, Hans-Christian Siebert, Günter Lochnit, Jens Kordelle, Markus Rickert, Jürgen Steinmeyer

**Affiliations:** 1 Department of Orthopedics, University Hospital Giessen and Marburg, Giessen, Germany; 2 RI-B-NT Research Institute of Bioinformatics and Nanotechnology, Kiel, Germany; 3 Department of Biochemistry, Justus-Liebig-University Giessen, Giessen, Germany; 4 Agaplesion Evangelical Hospital Mittelhessen, Giessen, Germany; University of Patras, Greece

## Abstract

Destruction of articular cartilage is a characteristic feature of osteoarthritis (OA). Collagen hydrolysates are mixtures of collagen peptides and have gained huge public attention as nutriceuticals used for prophylaxis of OA. Here, we evaluated for the first time whether different bovine collagen hydrolysate preparations indeed modulate the metabolism of collagen and proteoglycans from human OA cartilage explants and determined the chemical composition of oligopeptides representing collagen fragments. Using biophysical techniques, like MALDI-TOF-MS, AFM, and NMR, the molecular weight distribution and aggregation behavior of collagen hydrolysates from bovine origin (CH-Alpha®, Peptan™ B 5000, Peptan™ B 2000) were determined. To investigate the metabolism of human femoral OA cartilage, explants were obtained during knee replacement surgery. Collagen synthesis of explants as modulated by 0–10 mg/ml collagen hydrolysates was determined using a novel dual radiolabeling procedure. Proteoglycans, NO, PGE_2_, MMP-1, -3, -13, TIMP-1, collagen type II, and cell viability were determined in explant cultures. Groups of data were analyzed using ANOVA and the Friedman test (n = 5–12). The significance was set to p≤0.05. We found that collagen hydrolysates obtained from different sources varied with respect to the width of molecular weight distribution, average molecular weight, and aggregation behavior. None of the collagen hydrolysates tested stimulated the biosynthesis of collagen. Peptan™ B 5000 elevated NO and PGE_2_ levels significantly but had no effect on collagen or proteoglycan loss. All collagen hydrolysates tested proved not to be cytotoxic. Together, our data demonstrate for the first time that various collagen hydrolysates differ with respect to their chemical composition of collagen fragments as well as by their pharmacological efficacy on human chondrocytes. Our study underscores the importance that each collagen hydrolysate preparation should first demonstrate its pharmacological potential both *in vitro* and *in vivo* before being used for both regenerative medicine and prophylaxis of OA.

## Introduction

Collagen hydrolysates are mixtures of collagen peptides and are popular nutriceuticals used for prophylaxis of osteoarthritis (OA). Pharmacokinetic studies using mice showed that orally administered radioactive gelatine hydrolysates are resorbed and that some radioactivity is recovered within articular cartilage [1]. Collagen hydrolysates have been considered to be a safe food ingredient [2], [3]. Clinical investigations [4]–[6] with various preparations of collagen fragments consistently showed symptom-relieving effects of collagen hydrolysates, thus improving joint function and reducing joint pain. The methodological quality of those studies ranged from medium to very good [7]. Interestingly, a recently published pilot randomized controlled trial with 30 patients presenting with mild knee OA suggests that delayed gadolinium-enhanced magnetic resonance imaging of cartilage (dGEMERIC) was able to detect a change in the proteoglycan content of cartilage after 24 weeks in patients receiving a collagen hydrolysate formulation [8]. However, these preliminary data are of limited value due to the small sample size and missing morphometric MRI sequences [8]. The European Food Safety Authority (EFSA) panel on dietetic products, nutrition, and allergies recently concluded that so far, no cause-and-effect relationship between the maintenance of joints and the use of collagen hydrolysates has been shown [9].

Collagen hydrolysates have gained huge public attention initially due to an *in vitro* study published by Oesser et al. [10], in which collagen type I hydrolysates were found to stimulate the synthesis of proteoglycans and collagen using cultured healthy bovine articular chondrocytes [10]. Such an effect might antagonize the degradation and loss of matrix during progression of OA and even be useful for tissue engineering of cartilage [11]. However, using collagen type II fragments from human and bovine cartilage, increased matrix degradation and expression of MMPs as well as decreased collagen type II biosynthesis were discovered in cultured human and bovine chondrocytes or cartilage explants [12], [13]. These observations may either contribute to the OA destruction of cartilage or just be normal endogenous metabolic feedback [12], [13]. Another remarkable study was recently published in which a ligand-receptor interaction of small collagen fragments with integrin domains was described, which might serve as a molecular mode of action of collagen hydrolysates [14], [15].

Inflammation and catabolism are both associated with OA, and a broad range of mediators were identified in OA joints. Inflammatory cytokines, like interleukin-1ß (IL-1ß) and tumor necrosis factor-α (TNF-α), have been identified as stimuli for the biosynthesis of abnormal proteases and as inhibitors of the production of collagen and proteoglycans by articular cartilage [16], [17]. Also, other inflammatory mediators, like nitric oxide (NO) and prostaglandin E_2_ (PGE_2_), were reported to be involved in OA and to interact with the cytokine-induced pathways [17], [18]. For instance, PGE_2_ was reported to upregulate IL-1, MMP-13, and ADAMTS5, to inhibit synthesis but stimulate release of proteoglycans from OA cartilage and to contribute to synovial inflammation [16], [18], [19]. NO has been proposed as an important mediator of cartilage destruction. Excess production of NO has been associated with decreased synthesis of aggrecan, type II collagen, and IL-1-receptor antagonist as well as increased MMP activity *in vitro* [20], thereby mediating cartilage destruction.

Identification of agents able to interfere with catabolic and inflammatory pathways has long been a therapeutic goal for inhibiting or slowing down the degradation of cartilage [21]. Even though stimulated collagen biosynthesis by chondrocytes, as reported for collagen type I hydrolysate, would be desirable, this effect has been only observed in healthy young bovine chondrocytes [10]. Whether the OA cartilage of the elderly human patient does respond similarly is speculative, since several studies have already shown that the metabolism of OA cartilage and of older patients is subject to severe alterations [22]–[24]. Furthermore, the biosynthesis of collagen by bovine chondrocytes, as modulated by collagen hydrolysates, was measured by the incorporation of radioactive proline into overall proteins [10], which does not reflect the biosynthesis of collagen type II directly. Also, no information about collagen hydrolysates on other pathways being involved in OA destruction has been published so far.

Thus, the present *in vitro* study was designed to examine for the first time whether and to what extent collagen hydrolysates obtained from various sources differ with respect to their biological activities on human OA cartilage. Specifically, we were interested to see if collagen hydrolysates 1. modulate the synthesis of collagen type II from human OA knee cartilage, 2. differ with respect to their chemical composition, such as molecular weight distribution of peptides, and 3. possess the potential to induce proteoglycan and collagen loss from cartilage explants.

## Materials and Methods

### MALDI-TOF Mass Spectrometric Analysis

Collagen hydrolysates from bovine origin (RDH, Peptan™ B 5000; RDH-N, Peptan™ B 2000 from Rousselot SAS, Puteaux, France; CH-Alpha® from Gelita Health Products GmbH, Eberbach, Germany) were used for our experiments. Using MALDI-TOF mass spectrometry, the molecular weight distributions of peptides from collagen hydrolysates were estimated. Collagen hydrolysates (1 mg/ml) were dissolved in water and precipitated with acetone overnight at −20°C, centrifuged at 4°C, and dried. Samples were redissolved in water, mixed (1∶1, v:v) with dihydroxybenzoic acid and methylendiphosphonic acid (5 mg/ml each) used as matrix solution, and subsequently applied onto the MALDI-TOF target as 1-µl droplets containing 1 µg collagen hydrolysate. Sample were allowed to crystallize. All mass spectra were acquired with a Bruker Ultraflex TOF/TOF MALDI instrument (Bruker Daltonics, Bremen, Germany) in the positive ion mode. The system utilizes a pulsed nitrogen laser, emitting at 337 nm. The “low mass gate” was set to open at m/z = 2200 for the linear mode and at m/z = 800 for the reflector mode. An extraction voltage of 25 kV was chosen. Each mass spectrum was obtained as an average from 128 single laser shots. The mean molecular weight was obtained in the linear mode and calculated from the m/z value lying in the middle between the m/z values of the ascending and descending peak, reaching 50% of the peak maximum.

### Atomic Force Microscopy (AFM)

AFM allows the visualization of molecular structures on a nanoscale size. With this technique, it is possible to determine if higher ordered structures, such as triple-helical collagen fragments, are present. AFM was performed in the tapping mode using an MFP-3D-Bio atomic force microscope (Atomic force, Germany). Collagen hydrolysates were dissolved in pure water at a concentration of 10 ng/mL, adsorbed onto mica discs (Plano, Germany), incubated for 15 min at room temperature, dried with nitrogen, and imaged under air in tapping mode with OMCL-AC240TS-W2 cantilevers (Atomic force). The data were processed using the MFP-3D interface built on IGOR PRO version 6.02A [14], [15].

### Nuclear Magnetic Resonance (NMR) Spectroscopy

One- and two-dimensional proton NMR spectra can be used to describe various collagen hydrolysates in respect to their characteristic chemical shift patterns. In order to identify the diverse proton resonance signals, which can be assigned by their ppm values to, for example, hydrogen atoms of Arg residues in the collagen fragments, two-dimensional NMR spectra are necessary. Two-dimensional NOESY (nuclear Overhauser spectroscopy) NMR experiments provide information about the magnetization transfer between hydrogen atoms that are nearby in space to each other. With two-dimensional TOCSY (total correlation spectroscopy) NMR experiments, one can analyze the magnetization transfer via the chemical bonds. In combination, both two-dimensional NMR techniques are sufficient for delivering characteristic fingerprints of the collagen hydrolysates under study.

The NMR samples (collagen hydrolysates) were dissolved at an amount of 3 mg in 0.5 ml water (90% H_2_O/10% D_2_O). The NMR experiments were performed on a 600 MHz Bruker Avance III spectrometer at 298K. 2D-TOCSY experiments (DIPSI-2; mixing time 80 ms) and 2D-NOESY (mixing times 200 or 400 ms) were recorded with 512 (F1)×1024 (F2) complex data points and a spectral width of 7212 Hz (12 ppm). Water suppression was performed using excitation sculpting, and 16 scans per increment were accumulated with an inter-scan recovery delay of 1.5 s. For processing, we used zero-filling to 1024 (F1)×2048 (F2) data points prior to Fourier transformation, followed by baseline correction in both dimensions. Spectra were calibrated on internal water.

### Specimen Selection

Articular cartilage was obtained in full thickness from the lateral femoral condyles of OA patients undergoing knee replacement surgery (collagen biosynthesis experiments: N = 12, age 65.9±9.8 y, BMI 31.7±5.9; cartilage degradation experiments: N = 5, age 61.0±13.4 y, BMI 30.5±4.3). The degree of OA changes of femoral condyles was subsequently determined according to Collins [25]. Four-millimeter-diameter cartilage discs were washed three times with Geýs balanced salt solution (GBSS) and subsequently cultured as described below. The time needed to process cartilage obtained from surgery to culture was always below 4 hours. OA patients were selected at random from our clinic. The use of human articular cartilage for this study was approved by the local ethics committee of Justus-Liebig-University of Giessen (Germany), and all patients provided written informed consent before starting the experiments.

### Articular Cartilage Explant Culture

Cartilage explants from mild (Collins grade <1.5) or moderately (Collins grade 1.5–3) affected lateral human OA condyles were cultured separately in 2.0 ml Ham's F-12, 2.5 mM HEPES (pH 7.2) containing 30 µg/ml alpha-ketoglutarate, 300 µg/ml glutamine, 50 µg/ml ascorbate, 1.0 mM Na_2_S0_4_, 20 units/ml penicillin, 10 µg/ml streptomycin, 2.5 µg/ml amphotericin B and 50 µg/ml gentamycin, 485 µg/ml CaCl_2_x2H_2_0 and 1% (v/v) CR-ITS^+^™ Premix (Collaborative Biomedical Products, Bedford, MD) [26]–[28]. Explants were cultured for 4–6 days in a normal bench top CO_2_-incubator under sterile conditions in order to first stabilize the cartilage metabolism at 37°C, 5% CO_2_, and 95% relative humidity.

The collagen biosynthesis was determined by isolating radioactive hydroxyproline after a novel dual radiolabeling procedure developed by Goodwin et al. [29] to minimize the heterogeneity among pairs of samples by using [^3^H]-proline as a baseline measurement and [^14^C]-proline incorporation during experimental treatment so that each explant has its own internal control. For dual radiolabeling, media were changed, explants were labeled for 24 h with 20 µCi/ml L-[2,3-^3^H]-proline (Perkin Elmer, Boston, MA) in the presence of 50 µg/ml freshly prepared ascorbic acid. Explants were washed several times in order to remove unincorporated [^3^H]-proline, as controlled by determination of radioactivity within wash solutions by liquid scintillation counting. Media were added, and cartilage explants were radiolabeled again for 24 h with 10 µCi/ml L-[^14^C(U)]-proline after addition of 50 µg/ml freshly made ascorbic acid in the presence of 0–10 mg/ml collagen hydrolysates. At the end of this second radiolabeling period, explants were washed three times with GBSS to remove unincorporated radioisotopes, as checked by liquid scintillation counting. Explants in GBSS and collected media were mixed with 10% (v/v) protease inhibitor cocktail (Complete™, Roche, Penzberg, Germany) according to the manufacturer’s instructions and stored frozen at −20°C until analysis.

In two separate sets of experiments, cartilage degradation was determined in the presence or absence of 5.0 ng/ml recombinant human interleukin-1ß (IL-1ß). Only lateral condyles of patients with cartilage of Collins grade 1,5–3 were used, and again, the metabolism of cultured explants was first stabilized for 4–6 days. In the first set of experiments, explants from 5 different patients were cultured for a period of 6 days in the presence of 0–10 mg/ml collagen hydrolysates with a media change after 3 days, and the loss of collagen, proteoglycans, matrix metalloproteinases (MMPs), NO, and prostaglandin E_2_ (PGE_2_) from explants into nutrient media was determined. In the second set of experiments, explants from 6 additional different patients received IL-1ß together with 0–10 mg/ml collagen hydrolysates and NO, and proteoglycan loss was evaluated during a culture period of 3 days. Media and explants were frozen at −20°C in the presence of proteinase inhibitors until analysis.

### Determination of Collagen Synthesis

Dual radiolabeled cartilage explants were thawed and washed three times with 1.0 ml GBSS. Excess liquid was removed by blotting the specimen on filter paper, and tissue wet weights were determined. Explants were dried at 30°C after treatment with acetone; the dry weight was determined and then hydrolyzed for 18–24 h at 108°C with 0.6 ml 6 N HCl. Radioactive hydroxyproline was assayed by using a method described by Juva and Prockop [30], as modified by Switzer and Summer [31]. Preliminary experiments revealed that unincorporated radioactive precursors were removed completely by this procedure. The radioactivity of both [^3^H]- and [^14^C]-labeled hydroxyproline oxidation products was normalized with respect to the dry weights of explants. [^3^H]-radioactivity was used as internal control by first calculating the (^14^C/^3^H) ratio of explants, as described by Goodwin et al. [29]. This ratio was then used to determine the percentage of collagen synthesis of treated explants versus untreated ( = 100%) controls. Thus, the untreated controls provide the basal ratio against which the treated explants are compared.

### Analysis of Collagen Loss

Collagen fragments released from the explants were determined in nutrient media using a commercially available collagen type II ELISA (MD Bioproducts, Zurich, Switzerland) according to the instructions provided by the manufacturer. Preliminary experiments revealed that none of the collagen hydrolysates was detected by this ELISA, indicating that peptides were mainly derived from collagen type I. Data were normalized by the cartilage wet weight.

### Analysis of Proteoglycan Loss

Papain-digested cartilage explants and culture media were assayed for sulfated glycosaminoglycans (GAGs) by reaction with 0.25 ml 1,9-dimethylmethylene blue dye solution (Serva, Heidelberg, Germany) in polystyrene 96-well plates and quantified by spectrophotometry at 523 nm using an ELISA plate reader. Chondroitin sulfate A from bovine trachea (Sigma, St. Louis, MO) was used as the standard [28], [32]. Proteoglycan loss was then calculated from the ratio of GAGs found in the media to total GAGs found in media plus explants. Data were normalized by the cartilage wet weight.

### Determination of NO Production

Nitrite levels in media were measured in duplicate using the Griess reaction with sodium nitrite as standard as described earlier [27], [28], [33]. Briefly, nitrate, being present in 0.1 ml media, was first reduced at 37°C for 20 minutes using 10 µl nitrate reductase (0.4 U/ml, Roche Diagnostics GmbH, Mannheim, Germany). Media samples were then mixed with an equal volume of Griess reagent (1% sulfanilamide and 0.1% *N*-1-naphthylethylenediamine dihydrochloride in 25% (v/v) H_3_PO_4_) and incubated for 5 minutes at room temperature, and the optical density was measured at 523 nm in an ELISA photometer [27], [28], [33]. Data were normalized by the cartilage wet weight.

### Determination of PGE_2_, MMPs and TIMP-1

PGE_2,_ MMP-1, -3, and -13; and TIMP-1 within media were measured using commercially available ELISA kits (PGE_2_-kit from Cayman, Ann Arbor, MI; MMP-1-kit from Calbiochem, Darmstadt, Germany; TIMP-1-, MMP-3- and MMP-13-kit from GE Healthcare, Little Chalfont, UK) according to the instructions provided by the manufacturers. Preliminary experiments revealed that none of the collagen hydrolysates tested interfered with the ELISAs used. Data were normalized by the cartilage wet weight.

### Chondrocyte Viability

The viability of chondrocytes within each of the anatomical zones of cartilage explants (superficial, intermediate, and deep layer) was determined in slices of separately cultured explants treated with or without 10 mg/ml collagen hydrolysates using the fluorescent probes fluorescein diacetate (Sigma, St. Louis, MO) and propidium iodide (Sigma, St. Louis, MO) as described previously [28], [34]. Briefly, at the end of the experiments, cartilage explants were washed twice with sterile isotonic phosphate-buffered saline (PBS). Tissue sections were then cut perpendicular to the joint surface, each being 100 µm thick. Cartilage slices were placed in a small drop of sterile PBS on a glass slide; 200 µl Ham’s F-12 media containing 0.1 mM fluorescein diacetate and 0.3 mM propidium iodide were added and subsequently incubated in the dark at 37°C and 95% humidity for 5 min. At two sites of the 3 slices per explant, at least 50 viable and/or dead cells, as indicated by green or red fluorescence, were counted in each of the 3 cartilage layers (superficial, intermediate, and deep zone) under a fluorescence microscope at 200-fold magnification.

### Statistical Analysis

Each experimental condition was repeated four (cartilage degradation), five (cartilage degradation in the presence of IL-1ß), or 11 times (collagen synthesis) using explants obtained from 5, 6, or 12 different patients, respectively (n = 5, 6 or 12). The data obtained from treated explants were compared with values from untreated controls from the same joint. Groups of data were evaluated using one-way analysis of variance (ANOVA) and the Friedman test. Data presented are mean±standard deviation, and the significance was set to p≤0.05.

## Results

### Biochemical Characterization of Collagen Hydrolysates

**Figure 1:**
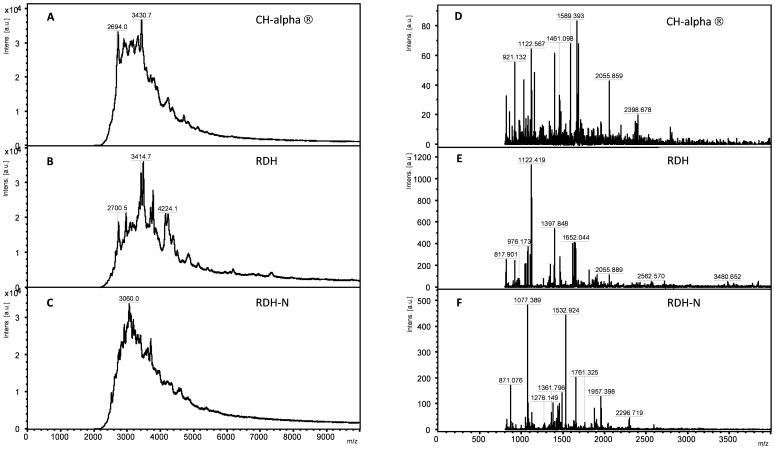
Compositional differences of collagen hydrolysates as determined by MALDI-TOF-MS. Mass spectra obtained in the (**A–C**) linear and (**D–F**) reflector mode reveal differences between (**A,D**) CH-Alpha®, (**B,E**) RDH, and (**C,F**) RDH-N with respect to peptide composition, as represented by the molecular weight distribution of the peptides, and the average molecular weight of each collagen hydrolysate preparation.

MALDI-TOF-MS analysis revealed qualitative differences between collagen hydrolysates obtained from different sources with respect to peptides identified in each preparation, width of molecular weight distribution, and the average molecular weight. Peaks identified from mass spectra obtained from both the linear (Figure 1A–C) and reflector (Figure 1D–F) modes clearly showed marked differences in the composition of peptides between collagen hydrolysates tested. The molecular weight distribution of peptides and the average molecular weight obtained from mass spectra in the linear mode for RDH (3,500 Da) were somewhat larger than those obtained from RDH-N (3,250 Da) and CH-Alpha® (3,300 Da) (Figure 1A–C).

**Figure 2:**
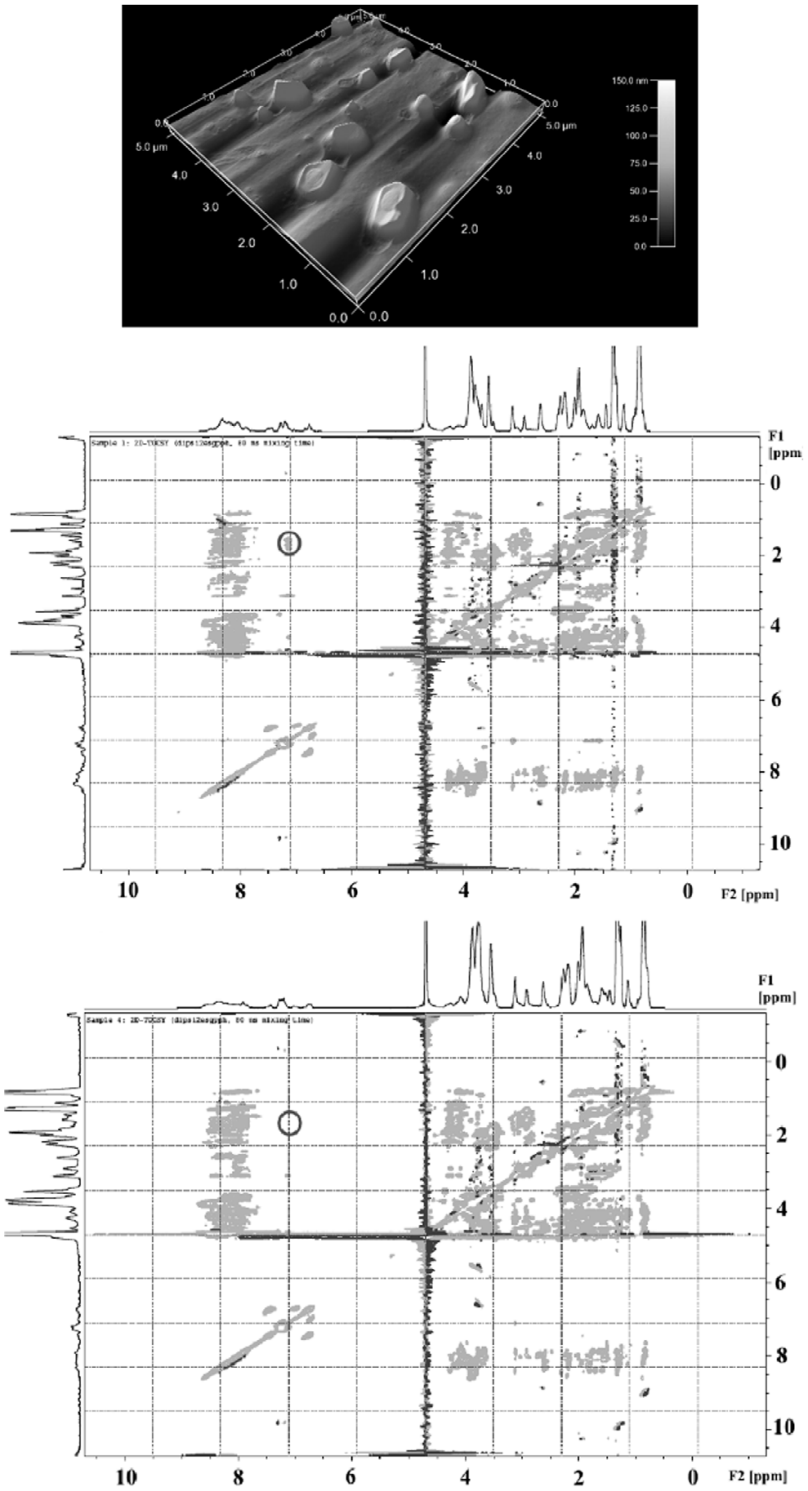
Three-dimensional AFM picture (top) and NMR-TOCSY spectra (middle, bottom) of collagen hydroylsates. The AFM picture (Fig. 2 top) shows the amorphous crystal-like structures in the presented 25 square micrometer area (0 to 5 micrometer) with a high resolution in the third dimension (0 to 150 nanometer), demonstrating that no higher-ordered collagen structures (eg, triple-helical collagen fragments) are present in the collagen hydrolysate preparation (RDH) from Rousselot. The highly resolved proton-NMR signals are displayed along the F1 and the F2 axes according to their ppm values (Fig. 2 middle and bottom). F1 is frequency axis one, F2 is the second frequency axis. The ppm values on both frequency axes correspond to the ppm values of the one-dimensional NMR spectrum. Depending on the relation of the magnetization transfer (via the bonds: TOCSY, via space: NOESY), an assignment of the signals to the protons is possible and thus leads to a characteristic fingerprint pattern of the collagen hydrolysate investigated (Fig. 2 middle and bottom). The TOCSY spectra (Fig. 2 middle and bottom) show, for example, one obvious difference in their signal patterns–namely, the characteristic cross-peak at F1: 1.5 ppm/F2: 7.2 ppm, highlighted as a grey circle in the TOCSY spectrum of RDH (Fig. 2 middle), is missing in the TOCSY spectrum of RDH-N (Fig. 2 bottom). This observation makes it clear that the fragments that belong to this cross-peak are missing in the sample RDH-N (Fig. 2 bottom). Thus, one change in the cross-peak signal pattern is already enough to distinguish between different collagen hydrolysates.

**Figure 3:**
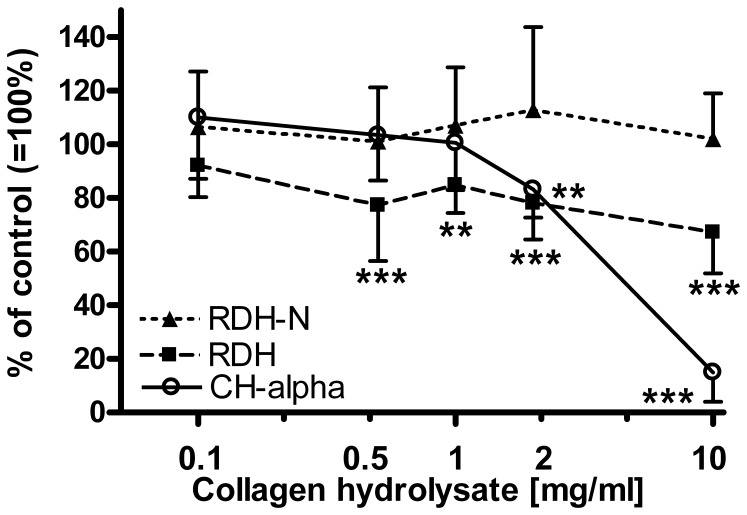
Effect of collagen hydrolysates on the collagen synthesis of OA cartilage. Incorporation of [^3^H]-proline and [^14^C]-proline into collagen of human cartilage explants was determined by measuring the radioactivity found in hydroxyproline. The [^14^C/^3^H]-incorporation ratio was then calculated and is expressed as percent of untreated control (100%) in the presence of 0.1, 0.5, 1, 2, and 10 mg/ml of RDH, RDH-N, or CH-Alpha®. Each experiment was done with explants removed from the lateral condyle of 6 patients graded with a Collins score of <1.5 and another 6 patients who presented lateral condyles with a Collin score between 1.5 and 3. Thus, explants from a total of 12 patients were used. Data shown are the mean±standard deviation (N = 12). Statistically significant different from untreated controls: **0.001<p≤0.01, ***p≤0.001.

AFM-analysis, in combination with NMR spectroscopy, revealed information about the aggregation behavior of the fragments from the collagen hydrolysates studied. RDH occurred in a smooth unstructured form, although some amorphous crystal-like structures were visible (Figure 2 - top). When comparing the TOCSY-spectrum of RDH with that of RDH-N, it was obvious that in the RDH-N TOCSY-spectrum, some signals were missing (Figure 2– middle and bottom). This observation clearly demonstrates that larger fragments present in RDH are removed or split off and that TOCSY experiments can be used as a fingerprint method to identify even complex collagen hydrolysates. The collagen fragments of RDH, RDH-N, and CH Alpha® analyzed with 2D-TOCSY NMR experiments [15] together with DOSY-NMR experiments [15] had a size range between 3.1–12.3 kDa (Figure 3 - middle), 0.4–3.3 kDa (Figure 2 - bottom), and 2.9–8.1 kDa, respectively.

### Effect of Collagen Hydrolysates on Collagen Synthesis

Even though the metabolism of cartilage from early stages of OA disease is known to differ from those of late stages [e.g. 35,36], we found that the response of early OA cartilage (Collins score <1.5) to treatment with the three collagen hydrolysates was statistically similar to cartilage with moderate OA alterations (Collins score 1.5–3). Thus, data obtained for collagen synthesis from cartilage with mild and moderately OA changes were combined to n = 12, and statistical analysis was performed.

Figure 3 shows that RDH and CH-Alpha® inhibited collagen synthesis concentration-dependently. However, a statistically significant inhibition was seen only for concentrations at 0.5 mg/ml for RDH and 2.0 mg/ml for CH-Alpha®, as well as at higher concentrations.

### Effect of Collagen Hydrolysates on MMPs and TIMP-1

**Figure 4:**
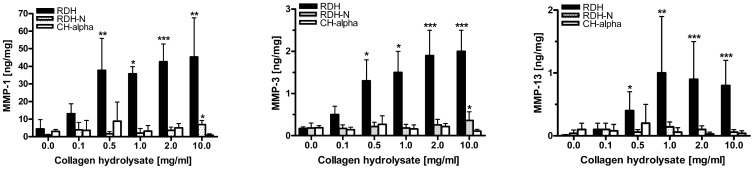
Concentration-dependent effect of collagen hydrolysates on the synthesis and/or release of MMP-1, MMP-3, and MMP-13. MMPs were determined within nutrient media of cultured human articular cartilage. Following stabilization of explant metabolism for 4–6 days, explants were treated for additional 6 days with 0–10 mg/ml collagen hydrolysate. MMPs were determined with ELISA, and data are expressed as mean±standard deviation (N = 5). Statistically significant different from untreated controls: *0.01<p≤0.05, **0.001<p≤0.01, ***p≤0.001.

Since collagen type II fragments isolated from human OA cartilage were reported to induce catabolic metabolism [12], [13], we also investigated whether collagen hydrolysates induce similar effects. RDH was found to markedly and dose-dependently increase the levels of MMP-1, -3, and -13 within nutrient media by up to 40-fold, 12-fold, and 125-fold, respectively (Figure 4). RDH-N was less active, in that a significantly increased amount of MMP-1 (6-fold) was found only at a high concentration of 10 mg/ml. The TIMP-1 levels remained unaffected by RDH and RDH-N but were decreased in the presence of 10 mg/ml CH-Alpha® (data not shown).

### Effect of Collagen Hydrolysates on Collagen and Proteoglycan Loss

In order to determine whether collagen fragments can induce degradation of extracellular matrix, OA cartilage explants were treated with RDH, RDH-N, or CH-Alpha®. Collagen hydrolysates, even when tested at a high concentration of 10 mg/ml or in the presence of IL-1ß, did not induce any elevated loss of collagen or proteoglycans from explants into nutrient media (data not shown).

### Effect of Collagen Hydrolysates on NO and PGE_2_

**Figure 5:**
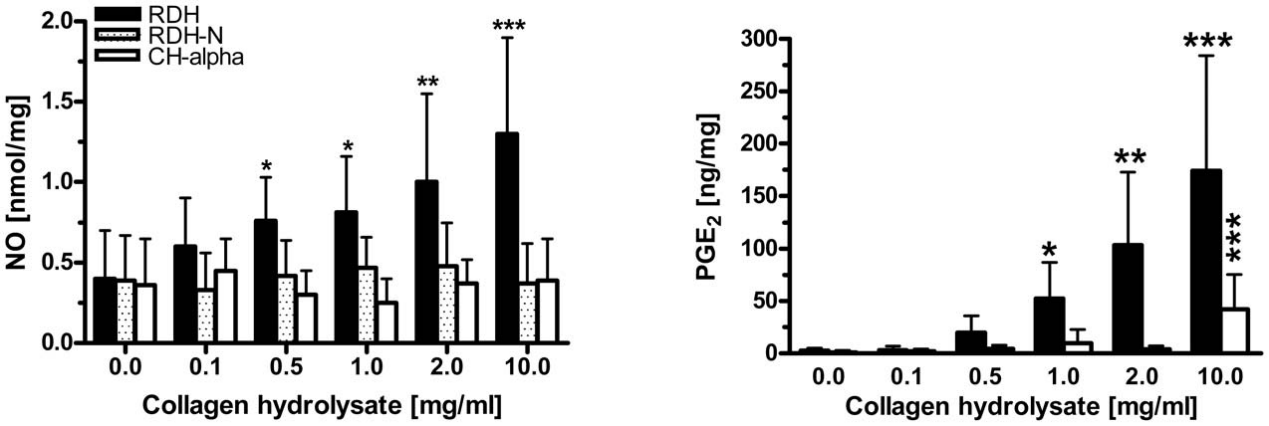
Concentration-dependent effect of collagen hydrolysates on the synthesis and/or release of NO and PGE_2_. ELISA was used to determine NO and PGE_2_ in media of cultured articular cartilage explants treated with 0–10 mg/ml collagen hydrolysates. Data are expressed as mean±standard deviation (N = 5). Statistically significant different from untreated controls: *0.01<p≤0.05, **0.001<p≤0.01, ***p≤0.001.

We next investigated the generation of PGE_2_ and NO, important inflammatory mediators affecting proteoglycan and collagen metabolism. Analysis of the NO secreted from treated explants into nutrient media revealed that only RDH dose-dependently augmented the content of NO (Figure 5). Furthermore, 1 mg/ml or higher concentrations of RDH as well as 10 mg/ml CH-Alpha®, but not RDH-N, induced significantly elevated levels of PGE_2_ within media (Figure 5). The collagen hydrolysates that were tested did not further enhance the level of NO in media of IL-1ß-treated explants (data not shown).

### Effect of Collagen Hydrolysates on the Viability of Chondrocytes

In order to exclude the possibility that the results obtained were due to impaired chondrocyte viability, cartilage sections were stained with propidium iodide and fluorescein diacetate, and subsequently, viable and dead cells were counted. Analysis of sections revealed that none of the collagen hydrolysates tested at the high concentration of 10 mg/ml induced any cytotoxic effects on chondrocytes of the superficial, intermediate, or radial zones of explants (data not shown).

## Discussion

Besides extensive marketing of nutraceuticals, only limited research about the efficacy, safety, and mechanisms of action has been performed, which is partly due to the minimal legal regulations when compared to the high statutory requirements imposed onto drugs. Our study is focused on collagen hydrolysates, a nutraceutical that has enjoyed large public attention for several years. We show for the first time that (1) the collagen hydrolysates RDH, RDH-N, and CH-alpha® differ with respect to both the molecular weight distribution of collagen fragments and by their biological activities on human chondrocytes. We demonstrate also for the first time that (2) none of the bovine collagen hydrolysates tested had any stimulatory effect on the biosynthesis of collagen by human articular knee cartilage and (3) that only RDH induces catabolic factors, like NO, PGE_2_, and MMPs, without resulting in any elevated degradation of the extracellular matrix.

Our mass spectrometric analysis as well as NMR experiments enabled us to show for the first time the different characteristic composition and structural organization of the investigated bovine collagen hydrolysates induced by various hydrolysis techniques [14], [15], [37]. Furthermore, we report for the first time about the different biological activities of collagen hydrolysates on human articular chondrocytes, which might be due to different active ingredient(s) of various collagen hydrolysate preparations, as shown by our biophysical analysis. These active ingredients might be either one or several oligopeptides or aggregates and remain to be determined.

The present study was carried out in order to understand the molecular mechanisms of the possible structure-modifying effect of collagen hydrolysates, as has been insinuated both in an animal model of OA and in a recently published clinical trial [8], [38]. Our study is the first to provide an *in vitro* demonstration of the lack of stimulatory effect of three bovine collagen hydrolysates on the biosynthesis of collagen by human OA knee cartilage. This observation was even independent of the degree of OA alterations, as found for cartilage explants. We investigated a broad range of concentrations, including those used previously [10] and even those being as high as 10 mg/ml, which are unlikely to be reached or accumulated *in vivo* within the joints. Moreover, collagen hydrolysates were used at concentrations that were thought to cover the assumed therapeutic plasma levels, which are currently unknown and which might lie below 1 mg/ml.

Our findings are in contrast to one previous study in which bovine collagen hydrolysate was reported to stimulate collagen biosynthesis by young bovine chondrocytes [10]. This discrepancy may be due to differences in the analytical methods applied, species, and age and health status of joints, which is in line with previous studies reporting severe alterations in the metabolism of cartilage due to age and OA [22]–[24]. In our study, we applied a novel dual proline radiolabeling technology to reduce the heterogeneity of biosynthetic data obtained when human cartilage explants are investigated [29]. The determination of the rate of radioactive proline incorporation without specific separation of collagens from overall proteins does not reflect the true rate of collagen synthesis, since the enrichment of proline in collagen as compared with other proteins is not assigned [39]. Therefore, we analyzed dual radiolabeled hydroxyproline to ensure that only the biosynthesis of collagen type II exclusively was determined [29], [30], [31]. Approximately up to 90% of total collagen in cartilage is of type II, which is considered, together with type IX, X, and XI, typical for cartilage and forms the backbone of cartilage heteropolymeric fibrils.

Cartilage destruction, mediated by the loss of collagen type II and proteoglycans, is a characteristic feature of OA [40], [41]. Using human cartilage from autopsy and arthroplasty, it was shown that during OA and aging, the initial damage to collagen type II occurs in the superficial and upper middle zones and extends into the deeper zones with increasing destruction. The initial injury is seen around chondrocytes in the pericellular region, and OA cartilage exhibits increased levels of cleaved and denatured collagen type II [40], [41]. Degradation of collagen type II is primarily mediated by MMP-13 but also by MMP-1 and -3, whereas loss of proteoglycans has been directly correlated with IL-1-induced expression of ADAMTS-4 and -5, followed by further enzymatic digestion by MMP-3, for example [42]. Currently, four different TIMP species have been identified, and only TIMP-3 has been shown to possess the potential to inhibit both ADAMTS-4 and -5 [43], [44]. Even though we found that RDH can induce elevated levels of MMP-1, -3, and -13 without changing the level of TIMP-1, this was not mirrored by any elevated loss of proteoglycans or collagen. Our findings indicate induction of TIMPs other than TIMP-1 like TIMP-3, absence of proteolytic processing of proMMPs to active MMPs and/or no effect of collagen hydrolysates on the expression of the major aggrecanases ADAMTS-4 and/or ADAMTS-5.

In order to avoid the possibility that we might have overlooked any pharmacological effect, we (a) used serum-free culture conditions to prevent the presence of serum growth factors and cytokines so that the responses solely due to collagen hydrolysates were identified; (b) sorted our explants removed from the same anatomical position according to the macroscopically visible pathological changes; and (c) cultured them in the presence of collagen hydrolysates over an extended period of six days. This approach is in line with the current difficulty in obtaining a comprehensive idea of the metabolic changes in OA due to the diversity in gene expression [45], [46]. This diversity may stem from regional differences of cellular metabolism and also from more or less OA-affected cartilage.

However, there are other components of cartilage breakdown that need to be further assessed. In this line, we also investigated whether collagen hydrolysates modulate the production respectively release of NO and PGE_2_ and may thus alter the PG synthesis and release using human explants [19]. RDH was found to significantly induce the production of PGE_2_ in moderately OA-affected cartilage. Attur et al. [19] reported that the predominant effects of PGE_2_ in OA chondrocytes are catabolic, in that PGE_2_ inhibits proteoglycan synthesis and stimulates matrix degradation in OA chondrocytes via the EP4 receptor. Even though RDH significantly increased the PGE_2_ level in our explant cultures, we observed no higher loss of proteoglycan and collagen. Increased NO synthesis has been associated with upregulated MMP activity, as has been also determined in our experiments [18]. However, the RDH-induced raised NO level does not correlate with any elevated loss of proteoglycans, which might be due to unaltered expression and/or activity of ADAMTS-4 and -5. These aggrecanases have to cleave aggrecan first before MMPs can further digest this proteoglycan.

In conclusion, our study has clearly elaborated for the first time that collagen hydrolysates from various sources differ significantly with respect to both their chemical composition of oligopeptides representing collagen fragments as well as their effects on human articular cartilage. Since marked effects on human chondrocytes were observed, depending on the collagen hydrolysates preparation investigated, our *in vitro* study indicates that collagen hydrolysates used as nutriceuticals might be either ineffective or even detrimental to OA cartilage. However, metabolized collagen fragments or other collagen hydrolysate preparations might contain therapeutically useful peptides. Thus, their biomedical properties have to be studied thoroughly both *in vitro* and in animal as well as clinical trials before being applied as safe and effective nutriceuticals in patients.
